# The effect of antenatal depression on birth weight among newborns in South Gondar zone, Northwest Ethiopia: a population-based prospective cohort study

**DOI:** 10.1186/s13690-021-00643-y

**Published:** 2021-07-05

**Authors:** Getnet Mihretie Beyene, Telake Azale, Kassahun Alemu Gelaye, Tadesse Awoke Ayele

**Affiliations:** 1 Department of Psychiatry, College of Health Sciences, Debre Tabor University, Debre Tabor, Ethiopia; 2 Department of Epidemiology and Biostatistics, College of Medicine and Health Sciences, University of Gondar, Gondar, Ethiopia; 3 Department of Health Education and Behavioral Sciences, College of Medicine and Health Sciences, University of Gondar, Gondar, Ethiopia

**Keywords:** Pregnancy, Depression, Antenatal depression, Birth weight, Ethiopia

## Abstract

**Background:**

There is a high prevalence of antenatal depression and low birth weight (LBW) (< 2.5 kg) in Ethiopia. Prior evidence revealed that the association between antenatal depression and LBW in high- and low-income countries is conflicting. The effect of antenatal depression on birth weight is under-researched in Ethiopia. We aimed to examine the independent effect of antenatal depression on newborn birth weight in an urban community in Northwest Ethiopia.

**Methods:**

A total of 970 pregnant women were screened for antenatal depression in their second and third trimester of pregnancy through the use of the Edinburgh Postnatal Depression Scale (EPDS). A logistic regression model was used to adjust confounders and determine associations between antenatal depression and low birth weight. Information was collected on the birth weight of newborns and mother**’**s socio-demographic, anthropometric, obstetric, clinical, psychosocial, and behavioral factors.

**Results:**

The cumulative incidence of LBW was found to be 27.76%. The cumulative incidence of LBW in those born from depressed pregnant women was 40% as compared to 21% in none depressed. While considering all other variables constant, mothers who had antenatal depression were 2.51 (COR = 2.51 (95 CI: 1.87, 3.37)) more likely to have a child with low birth weight. After adjusting for potential confounders, antenatal depression in the second and third trimester of pregnancy (AOR = 1.92 (95% CI: 1.31, 2.81)) remained significantly associated with LBW. Mid-Upper Arm Circumference (MUAC) ≤21, lack of ANC follow up, and preterm births were also associated with LBW.

**Conclusion:**

This study showed that antenatal depression during the second and third trimester of pregnancy is associated with LBW of newborns and replicates results found in high-income countries. Linking early screening, detection, and treatment of antenatal depression into routine antenatal care could be essential to improve pregnancy outcomes.

## Background

The antenatal period is typically referred to as the time from the starting of pregnancy and ends with the onset of labor [1]. The scientific literature indicates that this is a time when a pregnant woman is vulnerable to affective disorders; specifically, these are common in mid and late trimesters [2, 3]. During late pregnancy, fear of childbirth and dysfunctional coping style is associated with emotional disturbance [4]. Pregnancy can intensify the susceptibility to the mental, physical, and psychological health of women and their fetuses [5]. Among mental health problems that occurred during pregnancy, depression is the most prevalent psychiatric disorder affecting pregnant women [3, 6].

The prevalence rate of depression during pregnancy has been significantly higher in developing countries than in developed ones [7]. There is increasing evidence that the rate of depression during pregnancy is two to three times higher in Low and Middle-Income Countries (LMICs) as compared to High-Income Countries (HICs) [8]. In Ethiopia’s context, the prevalence of *antenatal depression* varies across different parts of the region; it *ranges* between 11.8% [9] and 31.2% [10].

Depressive symptoms during pregnancy may have devastating effects, not only for the women but also for the birth outcome and family [11]. Pregnant women with depressive symptoms experience social dysfunction, are more emotionally withdrawn, express excessive concern about the pregnancy and their ability to parent [12]. Furthermore, likely of using tobacco, alcohol, drugs, and less likely to have adequate prenatal care are known to affect the fetus, the baby, and the mother herself [13–16]. Besides this pregnant women with depression may also develop increased risk of preeclampsia [17], diabetes [18], and operative deliveries [19]. Reports from LMICs showed that antenatal depression significantly increased the risk of prolonged labor [20].

There is evidence that indicates antenatal depression also has numerous adverse effects on neonatal health outcomes [21–23]. Among these adverse effects, low birth weight is the prominent cause of neonatal, infant, childhood morbidity, mortality, and developmental impairments worldwide [24, 25].

Currently, different organizations are working to reduce LBW globally by 30% by the year 2025 [26]. Despite this activity, more than 20 million infants were born with LBW in 2015, almost half of them in Southern Asia and one quarter in Africa, with the majority born in Eastern and Western Africa [27]. Similarly, in Ethiopia, globally recommended strategies have been implemented. However, according to Ethiopia demographic and health Survey report, LBW is increasing from 11% in 2011 [28] to 13% in 2016 [29], and a variety of studies were conducted to estimate the prevalence of LBW in Ethiopia; ranges from 6.1% [30] to 29.1% [31]. There is spatial clustering of LBW in Ethiopia, specifically in the Amhara region [32],which is the most cause of early neonatal deaths [33, 34] and accounted for the highest case related neonatal mortality [35].

Studies from Australia [36], Finland [37], USA [23], and systematic review and a meta-analysis [38, 39] have reported that antenatal depression is associated with LBW. Few studies from LMICs such as Pakistan [21], India [40], China [41] and Malaysia [42] pointed out that antenatal depression was associated with an increased risk of LBW. In the Ethiopian context, there are only two studies one by Hanlon C.et al. [43] and the other by Fekadu DA et al. [44] that stated the effect of antenatal depression on birth weight, despite the high prevalence of antenatal depression [45, 46]. Of these, one is not specific to depression; instead, it stated the impact of antenatal common mental disorders upon perinatal outcomes [43].

We thought that antenatal depressive symptoms are independent of other factors that reduce the birth weight due to several behavioral features associated with depression: loss of motivation and interest in everyday activities [47],decreased healthy nutrition and increased unhealthy food [48], tendency to consume fewer macronutrients [49], inadequate maternal dietary intake [50] and elevated level of cortisol hormone [51] affect fetal growth and consequently lead to LBW.

Different study findings showed that improving psychosocial health facilities for antenatally depressed women in low-income communities can lead to improved neonatal outcomes [52]. Despite the improvement in health care services in Ethiopia, the status of LBW increases from time to time [28, 29]. This might be due to the recently reported high frequency of antenatal depression reported in a different region of Ethiopia. In this prospective community-based study, we addressed this shortcoming by examining the association between antenatal depression during the second and the third trimester of pregnancy and LBW neonates among urban women in Ethiopia.

## Methods

### Study design and settings

We conducted a community-based prospective cohort study at Debre Tabor and Woreta towns, which are situated in the South Gondar zone from June 2019 to March 2019 in the Northwest, Ethiopia.

According to the South Gondar zonal catchment profile, the estimated total population of Debre Tabor town was 84,382, of which 40,753 are females. While Woreta town has an estimated population of 41,668, of which 20,507 are females. About 2844 women at Debre Tabor and 1404 women in Woreta towns were estimated to be pregnant per year [53]. In these towns, there were one government-operated referral hospital, five health centers, and ten private health institutions providing health services during the data collection period.

Health extension workers (HEWs) are responsible for performing prevention and promotion activities, identifying and monitoring pregnant mothers, and maintaining up-to-date maternal records in each Kebele which is the lowest administrative unit or village in Ethiopia. The previous year’s report of the district health office showed that the proportion of pregnant women who were using antenatal care services at Debre Tabor and Woreta towns were estimated to be 75 and 64%, respectively [53]. Source population were all live new born from cohort pregnant women recruited at base line at Debre Tabor and Woreta towns and all live new born from cohort pregnant women at Debre Tabor and Woreta towns during the study period were used as study population.

### Sample size

The sample size was estimated using the double population formula, with the exposed and non-exposed ratio of 1:2 (exposed are those who are depressed and non-exposed are those who are not depressed). A 95% level of confidence, 80% power and 10% non-response rate were considered.

### Cohort recruitment and eligibility

All eligible and consenting women were recruited into the cohort. Pregnant women who were in their 2nd and 3^rd^trimesters, and living in Debre Tabor and Woreta Towns for at least the preceding 6 months, and without any cognitive or hearing impairment during the study period were included. Health Extension Workers (HEWs) identify all pregnant women in their respective kebeles and register them based on the criteria mentioned above.

The data collectors (HEWs) conducted an interview through the home-to-home visits to identify pregnant women who were willing to participate in this study and declared untraceable after three recruiting visits had been unsuccessful. All pregnant women who were aware of their pregnancy and fulfilled the criteria from June 2019 to October 2019 were included in the study. A total of 970 eligible women were identified and prospectively followed up until March 2020. Potentially newborn is eligible if they were born alive.

### Data quality management

Twenty experienced data collectors and two supervisors participated in the data collection process after 2 days of training. All the data collectors were recruited from each kebeles. The data collectors had a diploma and a degree in nursing. The supervisors had MSc educational level. The training was aimed to help trainees understand the contents of the questionnaire, objectives, and ethical issues relevant to the study. The ongoing quality of the data was closely monitored by supervisors and the authors of the study on a weekly face to face meeting and telephone communications.

### Measurement

#### Primary exposure

Antenatal depression was the primary exposure variable, which was assessed using the Edinburgh Postnatal Depression Scale (EPDS). The EPDS has a sensitivity and specificity of 78.9 and 75.3%, respectively, from a validation study in Ethiopia [54]. It includes ten items with a Likert scale of responses scored from 0 to 3 to a maximum score of 30, which was then coded as a categorical variable score ≥ 12 as indicative of the probable depressive disorder.

EPDS is a preferable scale than other depression scales to screen depression during pregnancy because it removes the physical symptoms of depression associated with pregnancy [55].

#### Outcome variables

The primary outcome variable was birth weight. Birth weight was categorized as low birth weight (< 2500 g) and normal birth weight (≥2500 g). The weight measurement of the babies was taken from a written paper (birth notification paper) with baby’s weight given from health facilities. If a mother did not have the paper the data collectors take the information from facilities records within 24 h of delivery. If the birth weight was not measured at health facilities, HEWs measured birth weight using Salter scales to the nearest 0.1 g.

#### Potential confounding variables

The most common potential confounding factors were considered in the present study based on previous literature such as threatening life events, social support, intimate partner violence, any chronic medical condition, ANC visit, history of complications current pregnancy, preterm delivery, history of alcohol consumption, malnutrition and socio-demographic and economic variables, including marital status, wealth index and level of education were also assessed.

Experiences of stressful life events during the 6 months before assessment were assessed using the List of Threatening Experiences (LTE). The scale contains twelve items and includes questions of death, illness, conflicts, and loss of property [56]. The presence of stressful life events explained by experienced one or more stressful life events in the last 6 months. The test-retest reliability of the LTE was good, with a Kappa of 0.61–0.87 and very good predictive validity [57].

The Oslo Social Support Scale (OSSS-3) [58] was used to measure maternal social support during pregnancy. The level of social support is classified as “poor support” 3–8, “moderate support” 9–11, and “strong support,” 12–14 scores. The OSSS-3 consists of three items assessing the number of close intimate, perceived level of concern from others, and perceived ease of getting helps from neighbors. The OSSS-3 has good convergent and predictive validity [59]. Both the LTE-12 and the OSSS-3 have been used in a population-level study in Ethiopia [60].

We identified women who were exposed to Intimate Partner Violence (IPV) during pregnancy by asking pregnant women three questions on emotional IPV, physical IPV, and sexual IPV. The presence of IPV was ascertained by the presence of at least one type of IPV [61].

The history of physician-diagnosed chronic medical conditions, including cardiac disease, hypertension was counted for each woman and recorded as “No” for those without any chronic medical conditions and “Yes” otherwise. The numbers of ANC visits were recorded. Preterm was categorized as Yes if babies were born alive before 37 weeks of pregnancy or No if otherwise. Stillbirth was considered as the death of the fetus after 20 completed weeks of gestation [62]. Gestational age was calculated based on the last normal menstrual period. However, gestational age was measured from ultrasound scan measurement, for women who were found to be unsure date of last normal menstrual period by linking them to the nearest health facility.

History of depression and family history of depression was assessed by using “case vignette”. History of current pregnancy complications: anemia, hypertension, edema, antepartum haemorrhage (APH) were counted for each woman and recorded as “yes/no.” Alcohol use was assessed using a four-item scale, the Fast Alcohol Screening Test (FAST) [63], which ranges from 0 to 16. Hazardous drinking refers to a score of three or more in a FAST scale [63].

The anthropometric indicators of the pregnant women used in the study were MUAC (in cm) to the nearest of 0.1 cm. MUAC is seen as a proxy indicator of the nutritional status of the women as well as it is relatively stable throughout pregnancy [64]; it is a validated and recommended measurement, with a cutoff score of 18 - 22 cm as underweight, and 22.1 - 31 cm as normal [65]. The wealth index was calculated using principal component analysis and categorized into three levels as High, Middle, and Low groups during ranking from the highest (usually the wealthiest) to the lowest scores (usually the poorest).

#### Analysis strategy

We used Stata software (version 14) for analysis. We run descriptive statistics and correlation of all the quantitative study variables. Percentage values, with their corresponding 95% confidence intervals (CIs), were used to summarize categorical variables. We used the odds ratio to measure the effect of antenatal depression on birth weight. We carried out Univariate logistic regression analyses for each risk factor to identify possible predictors associated with LBW. Variables with a *p* < 0.2 in the bivariate analyses were included in the multivariable logistic regression. The difference was deemed to be significant if *p* < 0.05. The total number of loss to follow up was 32 (3.2%). We also used a complete case analysis as it was suggested that less than 5% lost to follow up was with little concern [66, 67].

#### Ethical considerations

We obtained ethical approval from the Amhara region public health institute and the research ethics review committee of the University of Gondar. Before we want to collect the data participation information sheet was read for all participants and we obtained informed consent for all participants. Initially, pregnant women’s ≥ 2nd trimester was registered by health extension workers in all towns through home to home visits. Then, the sample size was allocated proportionally based on the actual number of registered pregnant women’s after having the list. Those women who were expressing suicidal ideation were referred to Debre Tabor referral Hospital Psychiatry unit for further diagnosis and appropriate management. The decision to refer was made by the project co-coordinator (GM) based on a review of all women who were expressing suicidal ideation.

## Results

**Fig. 1:**
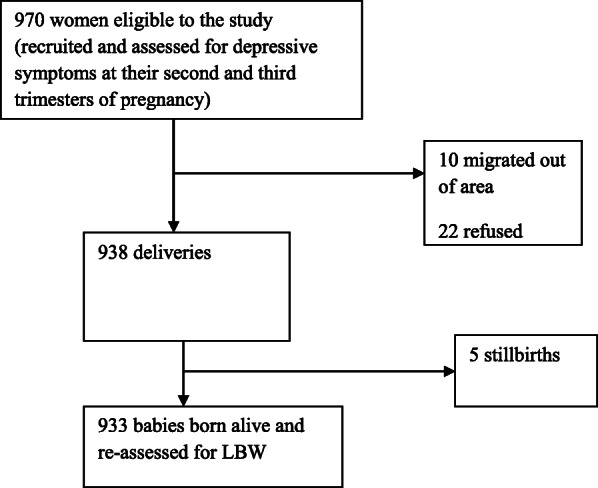
Flow chart of recruited pregnant women and outcome of birth weight

A flow chart of recruited women and the main outcomes is presented in Fig. 1. In total, 970 pregnant women were eligible to participate; 32 women (3.3%) were excluded during follow–up period from the sample for reasons that refusal to participate and moving from the area. Five pregnant women gave stillbirth. Finally, 933 women were included in the analysis, with a response rate of 96.2%.

The gestational age range from 16 to 34, with average gestation at recruitment was 25.6 weeks. Birth weight range was 1.8 kg to 3.9 kg, and with a mean birth weight (2.71 ± 0.45 SD). Two hundred and fifty-nine babies (27.76%) had a low birth weight i.e. < 2500 g.

### Socio-demographic characteristics of the study participants

According to chi square test, none of socio-demographics factors was significant. Out of 933 participants, 401(43%) were between the ages of 25–29 years. The mean (±SD) maternal age was 27.1 ± 4.8y. The data revealed that most of the participants were composed of young adults with 793(85%) formally educated, predominately at Diploma and above in educational level. The majority of women 864(92.6%) were Orthodox Christian by religion, and 624(92%) of the women were married (Table 1).

**Table 1:** Frequency distribution of Socio-demographic factors among pregnant women at Debre Tabor and Woreta towns, Northwest Ethiopia, 2020

Characteristics	Low birth weight		Total, n (%)	X^**2**^	***P***-value
	Yes, n (%)	No, n (%)			
**Age group**				1.4879	0.829
≤ 19	7 (2.70)	27 (4.00)	34 (3.64)		
20–24	69 (26.64)	167 (24.78)	236 (25.29)		
25–29	113 (43.63)	288 (42.73)	401 (42.98)		
30–34	49 (18.92)	129 (19.14)	178 (19.08)		
> 35	21 (8.11)	63 (9.35)	84 (9.00)		
**Religion**				2.1354	0.344
Orthodox	245 (94.59)	619 (91.84)	864 (92.60)		
Muslim	11 (4.25)	45 (6.68)	56 (6.00)		
Protestant	3 (1.16)	10 (1.48)	13 (1.39)		
**Ethnicity**				1.1724	0.279
Amhara	254 (98.07)	667 (98.96)	921 (98.71)		
Tigre	5 (1.93)	7 (1.04)	12 (1.29)		
**Education**				2.5674	0.633
Unable to read and write	32 (12.36)	85 (12.61)	117 (12.54)		
Able to read and write	7 (2.70)	16 (2.37)	23 (2.47)		
Primary	78 (30.12)	170 (25.22)	248 (26.58)		
High school	66 (25.48)	187 (27.75)	253 (27.12)		
Diploma and above	76 (29.34)	216 (32.05)	292 (31.30)		
**Occupation**				2.9230	0.571
Housewife	133 (51.35)	347 (51.48)	480 (51.45)		
Employee	71 (27.41)	164 (24.33)	235 (25.19)		
Merchant	35 (13.52)	112 (16.62)	147 (15.76)		
Student	8 (3.09)	14 (2.08)	22 (2.36)		
Daily laborer	12 (4.63)	37 (5.49)	49 (5.25)		
**Marital status**				2.3080	0.511
Single	16 (6.18)	31 (4.60)	47 (5.04)		
Married	232 (89.57)	624 (92.58)	856 (91.75)		
Widowed	5 (1.93)	9 (1.34)	14 (1.50)		
Divorced	6 (2.32)	10 (1.48)	16 (1.71)		
**Lack of food or Hunger**				0.0276	0.868
Yes	22 (8.49)	55 (8.16)	77 (8.25)		
No	237 (91.51)	619 (91.84)	856 (91.75)		
**Debt**				0.4981	0.480
Yes	29 (11.20)	65 (9.64)	94 (10.08)		
No	230 (88.80	609 (90.36)	839 (89.92)		
**Wealth Index**				1.1857	0.553
Low	84 (32.43)	228 (33.83)	312 (33.44)		
Middle	82 (31.66)	229 (33.98	311 (33.33)		
High	93 (35.91)	217 (32.19)	310 (33.23)		

### Obstetric and clinical characteristics of the respondents

Regarding the obstetric and clinical characteristics of the sample, approximately 533(57%) were found in the second trimester, and the remaining was in the third trimester, 109(12%) had a history of current pregnancy complication, 67(7%) had a chronic disease, 148(16%) experienced acute malnutrition (MUAC less than or equal to 21), 91(10%) experienced spontaneous preterm births, 6(0.64%) had twins delivery. Of the respondents, 374(40%) were primi-gravida with a mean of 1.9 per woman (Table 2).

**Table 2:** Frequency distribution of obstetric and clinical factors among pregnant women at Debre Tabor and Woreta towns, Northwest Ethiopia, 2020

Characteristics	Low birth weight			X^**2**^	***P***-value
	Yes, n (%)	No, n (%)	Total, n (%)		
**Unplanned pregnancy**				3.2763	0.070
Yes	110 (42.47)	243 (36.05)	353 (37.83)		
No	149 (57.53)	431 (63.95)	580 (62.17)		
**Pregnancy stage**				0.7960	0.372
Second trimester	154 (59.46)	379 (56.23)	533 (57.13)		
Third trimester	105 (40.54)	295 (43.77)	400 (42.87)		
**Number of live children(525)**				0.4114	0.814
One	70 (49.65)	181 (47.14)	251 (47.81)		
Two-four	68 (48.23)	192 (50.00)	260 (49.52)		
Five and above	3 (2.12)	11 (2.86)	14 (2.67)		
**History of current pregnancy complication**				11.2571	0.001
Yes	45 (17.37)	64 (9.50)	109 (11.68)		
No	214 (82.63)	610 (90.50)	824 (88.32)		
**History of abortion(*n*** **= 559)**				1.1700	0.279
Yes	29 (19.46)	64 (15.61)	93 (16.64)		
No	120 (80.54)	346 (84.39)	466 (83.36)		
**Modes of previous abortion (*n*** **= 93)**				0.8166	0.366
Spontaneous	25 (86.21)	59 (92.19)	84 (90.32)		
Assisted	4 (13.79)	5 (7.81)	9 (9.68)		
**Gravidity**				2.0214	0.364
One	110 (42.47)	264 (39.17)	374 (40.09)		
Two-four	128 (49.42)	366 (54.30)	494 (52.95)		
Five and above	21 (8.11)	44 (6.53)	65 (6.97)		
**Antenatal service**				48.9394	0.000
Yes	210 (81.08)	643 (95.40)	853 (91.43)		
No	49 (18.92)	31 (4.60)	80 (8.57)		
**Parity(544)**				0.9828	0.612
One	69 (47.26)	184 (46.23)	253 (46.51)		
Two-four	74 (50.69)	199 (50.00)	273 (50.18)		
Five and above	3 (2.05)	15 (3.77)	18 (3.31)		
**History of still birth(*n*** **= 559)**				0.2995	0.584
Yes	11 (7.38)	25 (6.10)	36 (6.44)		
No	138 (92.62)	385 (93.90)	523 (93.56)		
**Fear of pregnancy complication**				0.1373	0.711
Yes	138 (53.28)	350 (51.93)	488 (52.30)		
No	121 (46.72)	324 (48.07)	445 (47.70)		
**Chronic illness**				0.5417	0.462
Yes	16 (6.18)	51 (7.57)	67 (7.18)		
No	243 (93.82)	623 (92.43)	866 (92.82)		
**Twins**				9.3002	0.002
Yes	5 (1.93)	1 (0.15)	6 (0.64)		
No	254 (98.07)	673 (99.85)	927 (99.36)		
**Preterm**					
Yes	63 (24.32)	28 (4.15)	91 (9.75)	86.4770	0.000
No	196 (75.68)	646 (95.85)	842 (90.25)		
**Mid-upper arm circumference**					
< =21	70 (27.03)	78 (11.57)	148 (15.86)		
> 21	189 (72.97)	596 (88.43)	785 (84.14)		

### Psychosocial and behavioral characteristics of the study participants

Out of 933 participants, approximately 489(52.41%) experienced intimate partner violence, 416(45%) had poor social support, and 206 (22%) had hazardous level use of alcohol (Table 3).

**Table 3:** Frequency distribution of psychosocial and behavioral factors among pregnant women at Debre Tabor and Woreta towns, Northwest Ethiopia, 2020

Characteristics	Low birth weight		Total, n (%)	X^**2**^	***P***-value
	Yes, n (%)	No, n (%)			
**Depression**				38.4173	0.000
Yes	130 (50.19)	193 (28.64)	323 (34.62)		
No	129 (49.81)	481 (71.36)	610 (65.38)		
**Previous history of depression**				0.1891	0.664
Yes	74 (28.57)	183 (27.15)	257 (27.55)		
No	185 (71.43)	491 (72.85)	676 (72.45)		
**Family history of depression**				0.0016	0.968
Yes	36 (13.90)	93 (13.80)	129 (13.83)		
No	223 (86.10)	581 (86.20)	804 (86.17)		
**Life treating events**				15.9697	0.000
Yes	128 (49.42)	237 (35.16)	365 (39.12)		
No	131 (50.58)	437 (46.84)	568 (60.88)		
**Social support**				1.1504	0.563
Poor	117 (45.17)	299 (44.36)	416 (44.59)		
Moderate	92 (35.52)	224 (33.24)	316 (33.87)		
Strong	50 (19.31)	151 (22.40)	201 (21.54)		
**Intimate partner violence**				17.1062	0.000
Yes	164 (63.32)	325 (48.22)	489 (52.41)		
No	95 (36.68)	349 (51.78)	444 (47.59)		
**Alcohol use**				0.4520	0.501
Yes	61 (23.55)	145 (21.51)	206 (22.08)		
No	198 (76.45)	529 (78.49)	727 (77.92)		

### Factors associated with low birth weight

Bivariate regression analysis showed a significant positive association between LBW and antenatal depressive symptoms, a complication of current pregnancy, not ANC follow up, life-threatening events, intimate partner violence, MUAC≤21, preterm, and unplanned pregnancy. Multiple logistic regression analyses showed that, after adjustment for covariates, women with antenatal depression symptoms (AOR = 1.92 (95% CI: 1.31, 2.81)) were almost twice more likely to give LBW babies than women without antenatal depression symptoms. Other positively associated factors were babies born before 37 weeks (preterm), anthropometric measurement (MUAC), and lack of ANC follow up (Table 4).

**Table 4:** Bivariate and multivariable analysis for obstetric, clinical, and psychological factors among babies born alive at Debre Tabor and Woreta towns, Northwest Ethiopia, 2020

Characteristics	LBW		COR at 95% CI	AOR at 95% C	***P***-value
	Yes	No			
**Depression**					0.001
Yes	130	193	2.51 **(**1.87, 3.37**)**	1.92 (1.31, 2.81)*	
No	129	481	**1**	**1**	
**Complication of current pregnancy**					0.059
Yes	45	64	2.00 (1.33, 3.03)	1.58(.98, 2.54)	
No	214	610	**1**	**1**	
**Lack ANC follow up**					0.000
Yes	49	31	4.84 (3.01, 7.79)	3.91 (2.32, 6.59)**	
No	210	643	**1**	**1**	
**Life threatening events**					0.844
Yes	128	237	1.80 (1.35, 2.41)	1.04(.70, 1.54)	
No	131	437	**1**	**1**	
Mid upper arm circumference					0.000
< =21	70	78	2.83 (1.97, 4.06)	2.39 (1.60, 3.59)**	
> 21	189	596	**1**	**1**	
**Intimate partner violence**					0.586
Yes	164	325	1.85 (1.38, 2.49)	1.10(.77, 1.58)	
No	95	349	**1**	**1**	
**Preterm**					0.000
Yes	63	28	7.42 (4.62, 11.90)	6.68 (4.03, 11.08)**	
No	196	646	**1**	**1**	
**Unplanned pregnancy**					0.528
Yes	110	243	1.31(.98, 1.75)	1.11(.80, 1.54)	
No	149	431	**1**	**1**	

*Abbreviations*: *CI* Confidence interval, *OR* Odds ratio, *COR* Crude odds ratio, *AOR* Adjusted odds ratio;** (*P* < 0.001); *(*P* < 0.005); Hosmer-lemeshow: 0.2865

In the fully adjusted model: unplanned pregnancies (AOR = 1.11 (95%CI: 0.80, 1.54)), experience of threatening life events (AOR = 1.04; 95% CI 0.70, 1.54)),Complication of current pregnancy (OR = 1.58 (95% CI: 0.98, 2.54)),and intimate partner violence (AOR = 1.10 (95% CI: 0.77, 1.58)) during pregnancy were not associated with higher odds of low birth weight, even if there were strongly associated in bivariate analyses**.** No significant interaction between explanatory variables was found. A Hosmer-Lemeshow test indicated that the model fit the data well (*p* = 0.2865).

## Discussion

This is the second longitudinal-population-based study examining specifically the effect of antenatal depression on birth weight in our country. The main finding was that babies of women who had antenatal depression during second and third trimester of pregnancy increased the likelihood of giving birth to LBW children in Ethiopia.

While considering all other variables constant, mothers who had antenatal depression were 2.51 (COR = 2.51 (95 CI: 1.87, 3.37)) more likely to have a child with low birth weight. This association remains significant after adjustment for unplanned pregnancies, the experience of threatening life events, a complication of current pregnancy, intimate partner violence, no ANC follow up, preterm, and MUAC≤21,showing that antenatal depression has an independent effect on low birth weight.

The association that we have reported about the increased odds of low birth weight among women with antenatal depressive symptoms did not replicate the findings of the recent cohort studies from Ethiopia by Hanlon C.et al. [43] and Fekadu D A et al. [44], which were on the impact of antenatal common mental disorders upon perinatal outcomes and the effect of antenatal depression on adverse birth outcomes respectively. These conflicting results might arise from: One, both of the previous studies reveal that the proportion of babies with LBW were 7.1% [43] and 5.25% [44], which were less as compared to that observed in our study (27.76%), Pakistan 29% [68] and Nigeria14% [69] that underpowered to detect an association between antenatal depression and low birth weight. Second, the study done by Fekadu D A et al. excluded women with a severe symptoms of depression, which underestimated the effect of antenatal depression on the risk of LBW. Third, study done by Hanlon C.et al., was specifically on impact of antenatal common mental disorders upon perinatal outcomes which includes anxiety and somatic symptoms that is difficult to identify the independent effect of depression on birth weight and they used SRQ-20 includes several somatic items which could reflect physical symptoms of late pregnancy or physical ill-health rather than emotional distress. Moreover, the study in Butajira was studied among women with relatively economically well to do women compared to that of ours.

However, the positive effect of antenatal depression on the LBW that we have reported replicates results from the HICs [37] and LMICs [41]. Even if the exact biological mechanisms and interactions by which antenatal depression affects birth weight remain largely unknown, some research findings stated that antenatal depression stimulates the hypothalamus-pituitary-adrenal (HPA) axis leading to an increased cortisol hormone secretion specifically to the late pregnancy that restricts fetal growth [51]. In addition to this, women with antenatal depression have poor maternal dietary intake behavior and tend to consume fewer macronutrients, thereby leading to low birth weight [49, 50]. This collective mechanism speculates that antenatal depression plays an important role in the causation of intrauterine growth retardation and low birth weight, which in turn may lead to impaired mental development in infanthood and mental disorders later in life [70].

According to the EDHS report, the proportion of babies with LBW in our sample (27.76%) was double the estimated national average of LBW for Ethiopia [29]. These national estimates of LBW were largely derived from health facility data that cannot address babies born at home, which is likely to have led to an underestimation of underweight babies. Our prevalence estimate is similar to one of the few other community-based studies (28.3%) from Ethiopia [71].

It is well known that poor maternal nutritional status is the principal cause of LBW in low-income countries [72]. Mothers with MUAC less than ≤21 cm were two times more likely to give birth of low weight baby than mothers whose MUAC was more than 21. This result is congruent with studies conducted in other low-income countries [71, 73]. It may be speculated that LBW is associated with situations with uterine malnutrition due to alterations in placental circulation [72] and poor bioavailability of specific micronutrients such as oral vitamin B12 intake, possibly limiting fetal growth [74].

Antenatal care follow-up is essential for early identification and treatment of pregnancy-induced complications that lead to low birth weight newborns. Although we did not address the time of the first visit in this study, literature indicated that improving coverage of mental programs and early starting in ANC visit play a great role in improved birth weight [75, 76].

The odds of lack of ANC follow up among mothers who delivered LBW babies is about four times higher than those who had normal weight babies. The result is similar to those reported by other studies [31, 71]. Pregnant women who had no ANC follow-up, unable to early detect and treat pregnancy-induced complications that leads to LBW and may not receive routinely provision nutritional and lifestyle counseling during ANC visits that leads to better growth and development of the fetus [77].

This study also revealed that preterm neonates were significantly associated with LBW. Neonates delivered before 37 weeks of gestational age were more than six times more likely to have LBW as compared to term neonates. This study’s result is in line with the study conducted in Kenya [78],Tanzania [79],and other studies in Ethiopia Jimma [80] Gondar [81]. This might be as a result of intrauterine growth retardation and decreased skeletal muscle mass and subcutaneous fat tissue because of prematurity [76, 82].

### Limitations of the study

Our finding could be interpreted in the presence of some limitations. This study did not address the mechanisms by which antenatal depression affects the fetus. Evidence indicated that the effect of antenatal depression had been mediated by elevated antenatal depression norepinephrin levels [83]. Besides, we had no information on antidepressant medication for those referred to a psychiatric unit, thus could not assess their roles as a confounder in multivariable analysis. In addition to these, lack of data related to pre-pregnancy weight, dietary intake and preconception care is the limitation of this study.

## Conclusion

This population-based follow-up study found an independent association between antenatal depression in the second and third trimester of pregnancy and LBW in a sample of Ethiopian infants. Therefore antenatal depression has serious fetal consequences; early identification of depressive symptoms using the EPDS and treatment of antenatal depression could benefit not only maternal mental health but also the physical health, growth, and development of the fetus.

## Data Availability

The datasets supporting the conclusions of this article are available upon request to the corresponding author. Data were not publicly available to protect participant confidentiality.

## Abbreviations

ANC: Ante Natal Care
AOR: Adjusted Odds Ratio,
CI: Confidence Interval
COR: Crude odds ratio
OR: Odds Ratio
CM: Common Mental Disorder
EPDS: Edinburgh Postnatal Depression Scale
FAST: Fast Alcohol Screening Test
IPV: Intimate Partner Violence
LTE: List of Threatening Experiences
OSSS-3: Oslo-3 Social Support Scale
LBW: Low Birth Weight
MUAC: Mid Upper Arm Circumference

## References

[1] https://www.lawinsider.com/dictionary/antenatal-period.

[2] Evans J, Heron J, Francomb H, Oke S, Golding J. Cohort study of depressed mood during pregnancy and after childbirth. Bmj. 2001;323(7307):257–60. 10.1136/bmj.323.7307.257 PMC35345 11485953

[3] Fatoye FO, Adeyemi AB, Oladimeji BY (2004). Emotional distress and its correlates among Nigerian women in late pregnancy. J Obstet Gynaecol.

[4] Demyttenaere K, Lenaerts H, Nijs P, Van Assche FA (1995). Individual coping style and psychological attitudes during pregnancy predict depression levels during pregnancy and during postpartum. Acta Psychiatr Scand.

[5] Goebert D, Morland L, Frattarelli L, Onoye J, Matsu C (2007). Mental health during pregnancy: a study comparing Asian, Caucasian and native Hawaiian women. Matern Child Health J.

[6] Jafri SAM, Ali M, Ali R, Shaikh S, Abid M, Aamir IS (2017). Prevalence of depression among pregnant women attending antenatal clinics in Pakistan. Acta Psychopathol.

[7] Shah SMA, Bowen A, Afridi I, Nowshad G, Muhajarine N (2011). Prevalence of antenatal depression: comparison between Pakistani and Canadian women. JPMA.

[8] Organization WH. Maternal mental health and child health and development in resource-constrained settings: report of a UNFPA: World Health Organization; 2009.

[9] Bisetegn TA, Mihretie G, Muche T. Prevalence and predictors of depression among pregnant women. PLoS One. 2016;11(9). 10.1371/journal.pone.0161108 PMC5019395 27618181

[10] Sahile MA, Segni MT, Awoke T, Bekele D (2017). Prevalence and predictors of antenatal depressive symptoms among women attending Adama hospital antenatal clinic, Adama, Ethiopia. Int J Nurs Midwife.

[11] Alder J, Fink N, Bitzer J, Hösli I, Holzgreve W (2007). Depression and anxiety during pregnancy: a risk factor for obstetric, fetal and neonatal outcome? A critical review of the literature. J Matern Fetal Neonatal Med.

[12] Norbeck JS, Tilden VP. Life stress, social support, and emotional disequilibrium in complications of pregnancy: a prospective, multivariate study. J Health Soc Behav. 1983:30–46. 6853997

[13] Kahn RS, Certain L, Whitaker RC (2002). A reexamination of smoking before, during, and after pregnancy. Am J Public Health.

[14] Murray L, Cooper P, Hipwell A (2003). Mental health of parents caring for infants. Arch Womens Mental Health.

[15] Hanna EZ, Faden VB, Dufour MC (1994). The motivational correlates of drinking, smoking, and illicit drug use during pregnancy. J Subst Abus.

[16] Bonari L, Bennett H, Einarson A, Koren G (2004). Risks of untreated depression during pregnancy. Can Fam Physician.

[17] Lutsiv O, McKinney B, Foster G, Taylor V, Pullenayegum E, McDonald S (2015). Pregnancy complications associated with the co-prevalence of excess maternal weight and depression. Int J Obes.

[18] Spirito A, Ruggiero L, Coustan D, McGarvey S, Bond A (1992). Mood state of women with diabetes during pregnancy. J Reprod Infant Psychol.

[19] Chung TKH, Lau K, Yip ASK, Chiu HFK, DTS L (2001). Antepartum depressive symptomatology is associated with adverse obstetric and neonatal outcomes. Psychosom Med.

[20] Bitew T, Hanlon C, Kebede E, Honikman S, Fekadu A (2017). Antenatal depressive symptoms and perinatal complications: a prospective study in rural Ethiopia. BMC Psychiatry.

[21] Rahman A, Bunn J, Lovel H, Creed F (2007). Association between antenatal depression and low birthweight in a developing country. Acta Psychiatr Scand.

[22] Grigoriadis S, VonderPorten EH, Mamisashvili L, Tomlinson G, Dennis C-L, Koren G (2013). The impact of maternal depression during pregnancy on perinatal outcomes: a systematic review and meta-analysis. J Clin Psychiatry.

[23] Marcus SM. Depression during pregnancy: rates, risks and consequences. J Popul Ther Clin Pharmacol. 2009;16(1). 19164843

[24] Zerbeto AB, Cortelo FM, Élio FB (2015). Association between gestational age and birth weight on the language development of Brazilian children: a systematic review. J Pediatr (Versão em Português).

[25] Badshah S, Mason L, McKelvie K, Payne R, Lisboa PJ (2008). Risk factors for low birthweight in the public-hospitals at Peshawar, NWFP-Pakistan. BMC Public Health.

[26] Organization WH (2014). Organization WH: Global Nutrition Targets 2025: Low birth weight policy brief. Global Nutr Targets.

[27] Organization WH. UNICEF-WHO low birthweight estimates: levels and trends 2000–2015: World Health Organization; 2019.

[28] Macro: CSAaO (2011). Ethiopia demographic and health survey 2011.

[29] Macro: CSAaO (2016). Ethiopia demographic and health survey 2016.

[30] Dilnessa T, Belete E, Tefera M (2018). Prevalence of low birth Weight and associated factors among new born babies in Ataye primary hospital, north Shoa, Ethiopia. Asian J Med Health.

[31] Alemu T, Umeta M (2016). Prevalence and predictors of" small size" babies in Ethiopia: in-depth analysis of the Ethiopian demographic and health survey, 2011. Ethiop J Health Sci.

[32] Sisay MM, Muche AA. Spatial distribution and factors associated with low birth weight in Ethiopia using data from Ethiopian demographic and health survey 2016: spatial and multilevel analysis. bioRxiv. 2020. 10.1136/bmjpo-2020-000968 PMC8103935 34036183

[33] Desta M, Tadese M, Kassie B, Gedefaw M (2019). Determinants and adverse perinatal outcomes of low birth weight newborns delivered in Hawassa University comprehensive specialized hospital, Ethiopia: a cohort study. BMC Res Notes.

[34] Kahsay AH, Abebe HT, Gebretsadik LG, Tekle TH (2019). Survival and predictors of early neonatal death in neonatal intensive care unit of Mekelle general and Ayder comprehensive specialized hospitals, northern Ethiopia, 2018: prospective cohort study.

[35] Woldehanna T, Idejene E (2005). Neonatal mortality in a teaching hospital, North Western Ethiopia. Central Afr J Med.

[36] Eastwood J, Ogbo FA, Hendry A, Noble J, Page A, Group EYR (2017). The impact of antenatal depression on perinatal outcomes in Australian women. PLoS One.

[37] Räisänen S, Lehto SM, Nielsen HS, Gissler M, Kramer MR, Heinonen S. Risk factors for and perinatal outcomes of major depression during pregnancy: a population-based analysis during 2002–2010 in Finland. BMJ Open. 2014;4(11). 10.1136/bmjopen-2014-004883 PMC4244456 25398675

[38] Grote NK, Bridge JA, Gavin AR, Melville JL, Iyengar S, Katon WJ (2010). A meta-analysis of depression during pregnancy and the risk of preterm birth, low birth weight, and intrauterine growth restriction. Arch Gen Psychiatry.

[39] Jarde A, Morais M, Kingston D, Giallo R, MacQueen GM, Giglia L (2016). Neonatal outcomes in women with untreated antenatal depression compared with women without depression: a systematic review and meta-analysis. JAMA Psychiatry.

[40] Patel V, Prince M (2006). Maternal psychological morbidity and low birth weight in India. Br J Psychiatry.

[41] Li X, Gao R, Dai X, Liu H, Zhang J, Liu X (2020). The association between symptoms of depression during pregnancy and low birth weight: a prospective study. BMC Pregnancy Childbirth.

[42] Nasreen HE, Pasi HB, Rifin SM, Aris MAM, Ab Rahman J, Rus RM (2019). Impact of maternal antepartum depressive and anxiety symptoms on birth outcomes and mode of delivery: a prospective cohort study in east and west coasts of Malaysia. BMC Pregnancy Childbirth.

[43] Hanlon C, Medhin G, Alem A, Tesfaye F, Lakew Z, Worku B (2009). Impact of antenatal common mental disorders upon perinatal outcomes in Ethiopia: the P-MaMiE population-based cohort study. Tropical Med Int Health.

[44] Fekadu Dadi A, Miller ER, Woodman RJ, Azale T, Mwanri L (2020). Effect of antenatal depression on adverse birth outcomes in Gondar town, Ethiopia: a community-based cohort study. PLoS One.

[45] Bitew T, Hanlon C, Kebede E, Medhin G, Fekadu A (2016). Antenatal depressive symptoms and maternal health care utilisation: a population-based study of pregnant women in Ethiopia. BMC Pregnancy Childbirth.

[46] Tefera TB, Erena AN, Kuti KA, Hussen MA (2015). Perinatal depression and associated factors among reproductive aged group women at Goba and Robe Town of Bale Zone, Oromia Region, South East Ethiopia. Matern Health Neonatol Perinatol.

[47] Excellence NIfC. Depression in adults: recognition and management. NICE guideline (CG 90). Retreived from https://www.nice.org.uk/guidance/cg90. 2009.

[48] Barker ED, Kirkham N, Ng J, Jensen SK (2013). Prenatal maternal depression symptoms and nutrition, and child cognitive function. Br J Psychiatry.

[49] Payab M, ARD M, Eshraghian M, Rostami R, Siassi F, Ahmadi M (2012). The association between depression, socio-economic factors and dietary intake in mothers having primary school children living in Rey, South of Tehran, Iran. J Diabetes Metab Disord.

[50] Saeed A, Raana T, Saeed AM, Humayun A (2015). Effect of antenatal depression on maternal dietary intake and neonatal outcome: a prospective cohort. Nutr J.

[51] Diego MA, Field T, Hernandez-Reif M, Schanberg S, Kuhn C, Gonzalez-Quintero VH (2009). Prenatal depression restricts fetal growth. Early Hum Dev.

[52] Zimmer-Gembeck MJ, Helfand M (1996). Low birthweight in a public prenatal care program: behavioral and psychosocial risk factors and psychosocial intervention. Soc Sci Med.

[53] DDHO (2018). Debetabor District Health Office annual report on maternal and child health service performance.

[54] Tesfaye M, Hanlon C, Wondimagegn D, Alem A (2010). Detecting postnatal common mental disorders in Addis Ababa, Ethiopia: validation of the Edinburgh postnatal depression scale and Kessler scales. J Affect Disord.

[55] Cox JL, Holden JM, Sagovsky R (1987). Detection of postnatal depression: development of the 10-item Edinburgh postnatal depression scale. Br J Psychiatry.

[56] Brugha TS, Cragg D (1990). The list of threatening experiences: the reliability and validity of a brief life events questionnaire. Acta Psychiatr Scand.

[57] Montón-Franco C, Josefa G, Gómez-Barragán M, Sánchez-Celaya M, Ángel Díaz Barreiros M (2013). PsychometricpropertiesoftheListofThreateningExperiences—LTE anditsassociationwith psychosocialfactorsandmentaldisordersaccordingtodifferentscoringmethods. Affect Disord.

[58] DalgardOS DC, Lehtinen V, Vazquez-Barquero JL, Casey P (2006). Negativelifeevents, socialsupport and gender difference in depression: amultinational community survey with data from the ODINstudy. Soc Psychiatry Psychiatr Epidemiol.

[59] Boen H, Dalgard OS, Bjertness E. The importance of social support in the a ssociations between psychological distress and somatic health problems and socio-economic factors among older adults living at home: a crosssectional study. BMC Geriatr. 2012;12. 10.1186/1471-2318-12-27 PMC3464708 22682023

[60] FekaduA MG, SelamuM HM, AlemA (2014). Populationlevel mentaldistress inruralEthiopia. BMCPsychiatry.

[61] Rasch V, Van TN, Nguyen HTT, Manongi R, Mushi D, Meyrowitsch DW (2018). Intimate partner violence (IPV): the validity of an IPV screening instrument utilized among pregnant women in Tanzania and Vietnam. PLoS One.

[62] Wang H, Bhutta ZA, Coates MM, Coggeshall M, Dandona L, Diallo K (2016). Global, regional, national, and selected subnational levels of stillbirths, neonatal, infant, and under-5 mortality, 1980–2015: a systematic analysis for the global burden of disease study 2015. Lancet.

[63] Hodgson R, Alwyn T, John B, Thom B, Smith A (2002). The FAST alcohol screening test. Alcohol Alcohol.

[64] Organization WH (1995). Physical status: the use of and interpretation of anthropometry, report of a WHO expert committee: World Health Organization.

[65] M-t V, Antierens A, Sackl A, Staderini N, Captier V. Which anthropometric indicators identify a pregnant woman as acutely malnourished and predict adverse birth outcomes in the humanitarian context? PLoS Curr. 2013;5. 10.1371/currents.dis.54a8b618c1bc031ea140e3f2934599c8 PMC3682760 23787989

[66] Fewtrell MS, Kennedy K, Singhal A, Martin RM, Ness A, Hadders-Algra M (2008). How much loss to follow-up is acceptable in long-term randomised trials and prospective studies?. Arch Dis Child.

[67] V K, M M, P C (2004). Loss to follow-up in cohort studies: how much is too much?. Eur J Epidemiol.

[68] Jamshed S, Farah-Khan AB, Ali BB, Akram Z, Ariff M. Frequency of low birth weight and its relationship with maternal nutritional and dietary factors: a cross-sectional study. Cureus. 2020;12(6). 10.7759/cureus.8731 PMC7374259 32714671

[69] Ndu IK, Edelu BO, Uwaezuoke SN, Chinawa JC, Ubesie A, Ogoke CC (2015). J Neonatal Biol.

[70] Gale CR, Martyn CN (2004). Birth weight and later risk of depression in a national birth cohort. Br J Psychiatry.

[71] Assefa N, Berhane Y, Worku A (2012). Wealth status, mid upper arm circumference (MUAC) and antenatal care (ANC) are determinants for low birth weight in Kersa, Ethiopia. PloS One.

[72] Bale JR, Stoll BJ, Lucas AO. Reducing maternal mortality and morbidity. Improving birth outcomes: meeting the challenge in the developing world: National Academies Press (US); 2003. 25057689

[73] Adane T, Dachew BA (2018). Low birth weight and associated factors among singleton neonates born at Felege Hiwot referral hospital, north West Ethiopia. Afr Health Sci.

[74] Muthayya S (2009). Maternal nutrition & low birth weight-what is really important. Indian J Med Res.

[75] Gebremariam A (2005). Factors predisposing to low birth weight in Jimma hospital south western Ethiopia. East Afr Med J.

[76] Ramakrishnan U (2004). Nutrition and low birth weight: from research to practice. Am J Clin Nutr.

[77] Weight LB (1992). A tabulation of available information. Maternal Health and Safe Motherhood Programme.

[78] Muchemi OM, Echoka E, Makokha A. Factors associated with low birth weight among neonates born at Olkalou District Hospital, Central Region, Kenya. Pan Afr Med J. 2015;20(1). 10.11604/pamj.2015.20.108.4831 PMC4458305 26090056

[79] Siza J (2008). Risk factors associated with low birth weight of neonates among pregnant women attending a referral hospital in northern Tanzania. Tanzan J Health Res.

[80] Tema T (2006). Prevalence and determinants of low birth weight in Jimma zone, Southwest Ethiopia. East Afr Med J.

[81] Teshome D, Telahun T, Solomon D, Abdulhamid I (2006). A study on birth weight in a teaching-referral hospital, Gondar, Ethiopia. Central Afr J Med.

[82] Barton L, Hodgman JE, Pavlova Z (1999). Causes of death in the extremely low birth weight infant. Pediatrics..

[83] Glover V (1999). Mechanisms by which maternal mood in pregnancy may affect the fetus. Contemp Rev Obstet Gynecol.

